# Opinions about the most appropriate surgical management of diabetes-related foot infection: a cross-sectional survey

**DOI:** 10.1186/s13047-022-00523-w

**Published:** 2022-03-02

**Authors:** Leonard Seng, Aaron Drovandi, Malindu E Fernando, Jonathan Golledge

**Affiliations:** 1 Queensland Research Centre for Peripheral Vascular Disease, College of Medicine and Dentistry, James Cook University, 4811 Townsville, Queensland Australia; 2 Faculty of Health and Medicine, School of Health Sciences, University of Newcastle, Newcastle, New South Wales Australia; 3 Ulcer and wound Healing consortium (UHEAL), Australian Institute of Tropical Health and Medicine, James Cook University, Townsville, Queensland Australia; 4 Department of Vascular and Endovascular Surgery, Townsville University Hospital, Townsville, Queensland Australia

**Keywords:** Clinical practice, Diabetic foot disease, Orthopaedic surgery, Survey, Vascular surgery

## Abstract

**Background:**

There is a lack of high quality evidence to guide the optimal management of diabetes-related foot infection, particularly in cases of severe diabetes-related foot infection and diabetes-related foot osteomyelitis. This study examined the opinions of surgeons about the preferred management of severe diabetes-related foot infection.

**Methods:**

Vascular and orthopaedic surgeons in Australia and New Zealand were invited to complete an online survey via email. The survey included multi-choice and open-ended questions on clinical management of diabetes-related foot infection. Responses of vascular surgeons and orthopaedic surgeons were compared using non-parametric statistical tests. Open-text responses were examined using inductive content analysis.

**Results:**

29 vascular and 20 orthopaedic surgeons completed the survey. One-third (28.6%) used best-practice guidelines to assist in decisions about foot infection management. Areas for guideline improvement identified included more specific advice regarding the indications for available treatments, more recommendations about non-surgical patient management and advice on how management can be varied in regions with limited health service resource. The probe-to-bone test and magnetic resonance imaging were the preferred methods of diagnosing osteomyelitis. Approximately half (51.2%) of respondents indicated piperacillin combined with tazobactam as the preferred antibiotic choice for empirical treatment of severe diabetes-related foot infection. Negative pressure wound therapy was the most common way of managing a wound following debridement. All vascular surgeons (100%) made revascularisation decisions based on the severity of ischemia while most orthopaedic surgeons (66.7%) were likely to refer to vascular surgeons to make revascularisation decisions. Vascular surgeons preferred using wound swabs while orthopaedic surgeons favoured tissue or bone biopsies to determine the choice of antibiotic. Respondents perceived a moderate variation in management decisions between specialists and supported the need for randomised controlled trials to test different management pathways.

**Conclusions:**

Most vascular and orthopaedic surgeons do not use best-practice guidelines to assist in decisions about management of diabetes-related foot infection. Vascular and orthopaedic surgeons appear to have different preferences for wound sampling to determine choice of antibiotic. There is a need for higher quality evidence to clarify best practice for managing diabetes-related foot infection.

**Supplementary Information:**

The online version contains supplementary material available at 10.1186/s13047-022-00523-w.

## Background

Foot infection is a common complication of diabetes and varies in severity [1, 2]. Severe diabetes-related foot infection (DFI) often precipitates hospital admission and requirement for lower extremity amputation [3]. Management of DFI is challenging due to difficulties with diagnosis, limited evidence from high quality clinical trials and heterogeneity in clinical presentation [3]. Bone biopsy, for example, is the gold-standard method to diagnose diabetes-related foot osteomyelitis and to determine choice of antibiotic but is highly invasive and not always appropriate to use [3, 4]. International best-practice guidelines for management of DFI recognise that the evidence to support recommendation is limited due to the lack of high-quality clinical trials [3]. Only one reported randomised clinical trial has tested whether surgical or medical treatment is superior for treating diabetes-related foot osteomyelitis [5]. This trial was too small with too short follow-up to clarify the most appropriate management [5].

The lack of high quality evidence means that there is limited consensus to guide optimal management of DFI, particularly in cases of severe DFI and diabetes-related foot osteomyelitis [6–8]. This is echoed in a recent survey of Australian and New Zealand infectious diseases clinicians which reported limited consensus on how DFI was treated amongst this group of clinicians [9]. In Australia and New Zealand, surgical management of DFI is mainly performed by vascular and orthopaedic surgeons, but these specialties were not included in the previous survey on this topic [10, 11]. The aim of this study was to discover vascular and orthopaedic surgeons’ opinions about the management of DFI. Due to the prior evidence of variation in practice in Australia and New Zealand, the survey was focused on vascular and orthopaedic surgeons practicing in this region.

## Methods

### Study design

This descriptive cross-sectional study administered an online survey through the Qualtrics platform between January 2021 and April 2021. The survey was piloted and refined in consultation with vascular surgeons with experience in managing DFI. The final 21-question survey had three sections: Participant demographics (5 questions); management of DFI and osteomyelitis (10 questions); clinical consensus and areas for further research (6 questions). The survey included both multiple choice and free-text questions to facilitate in-depth responses. A full copy of the survey is included in Additional File 1. Likert scales (5-point rating scale) were used to gauge perceived usefulness of diagnostic modalities for osteomyelitis, variation in clinical practice, confidence in managing key aspects of DFI and perceptions on the need for further clinical trials.

### Survey dissemination

A purposive sampling technique was used to distribute the online survey. Professional associations whose members were vascular or orthopaedic surgeons and likely to be involved in the surgical management of DFI were approached to assist with disseminating the survey in November 2020. The associations were requested to distribute the survey link to their members in the first quarter of 2021, with those agreeing to assist in dissemination including Diabetes Feet Australia, Australian Orthopaedic Association, Australia and New Zealand Society for Vascular Surgery, the Queensland Statewide Diabetes Clinical Network and New South Wales Diabetes and Endocrine Network. Organisations did not send out repeat invitations to complete the survey, and the authors did not contact individual hospital departments or staff to complete the survey.

### Data analysis

Survey responses were deemed eligible for inclusion if at least 50% of the questions were completed. Descriptive analysis was conducted to determine participant characteristics and to sum their responses. The Mann-Whitney U and Yates Continuity Correction tests were used to assess statistical differences between the responses of the vascular and orthopaedic surgical specialties, and also between responses of surgeons working in the public and private setting. The Fisher-Freeman-Halton test was used to analyse contingency tables greater than 2 × 2 and where there were expected cell counts of less than 5. A p-value of < 0.05 was considered statistically significant. Statistical analyses were performed using SPSS V25 (IBM Corp, Armonk, NY, USA). Open-text responses were analysed using inductive content analysis, performed by LS, who read all responses and generated categories to provide a description of the responses. A second author (AD) reviewed and discussed these categories with the first author, with disagreements resolved by consensus when necessary. As not all survey respondents answered every question, the number of respondents answering a question was used as the denominator for the relevant results of that question and percentages calculated using this denominator.

## Results

### Participants

A total of 49 survey responses were received, with 42 being complete and seven including responses to at least 50% of questions. Participant characteristics are shown in Table 1. 29 responses (59.2%) were from vascular surgeons and 20 responses (40.8%) were from orthopaedic surgeons. Most (46; 93.9%) were Royal Australasian College of Surgeons accredited consultants.

**Table 1:** Characteristics of the forty nine participants

Surgical specialty Vascular surgery Orthopaedic surgery	29 (59.2%) 20 (40.8%)
**Location** Queensland New South Wales South Australia Tasmania Victoria Western Australia Australian Capital Territory New Zealand	29 (59.2%) 5 (10.2%) 2 (4.1%) 1 (2.0%) 4 (8.2%) 3 (6.1%) 2 (4.1%) 3 (6.1%)
**Primary place of work** Public hospital Private practice	31 (63.3%) 18 (36.7%)
**Years of medical experience**	27.0 (9.4)
**Designation** RACS accredited Consultant Others	46 (93.9%) 3 (6.1%)

Data presented as number (%) and mean (standard deviation). RACS = Royal Australasian College of Surgeons

### Current management practices

Responses to questions on current management practices are summarised in Table 2. The severity of infection was determined most commonly by the degree of tissue necrosis (30/49; 61.2%) and/or the extent of erythema (27/49; 55.1%). International classification systems were rarely used to determine the extent of infection (13/49; 26.5%). The most common wound sampling method for guiding the choice of antibiotic was the wound swab (27/49; 55.1%), followed by tissue or bone biopsy (17/49; 34.7%). There was a statistically significant difference (Fisher-Freeman-Halton = 14.512, *p* < 0.001) in the sampling method preferred by vascular and orthopaedic surgeons. Vascular surgeons (22/29; 75.9%) preferred wound swabs but orthopaedic surgeons (13/20; 65.0%) preferred tissue or bone biopsies to guide antibiotic choice for severe DFI. Most respondents (35/49; 71.4%) did not use a guideline to assist their management of DFI. This finding was consistent in both surgical specialties (x^2^ = 0.610, *p* = 0.435).

**Table 2:** Current management practices

	Total	Vascular surgeons	Orthopaedic surgeons	p-value
**Determining extent of infection prior to surgical treatment**^**1**^				
Based on international classification system	13/49 (26.5%)	9/29 (31.0%)	4/20 (20.0%)	^2^
Based on extent of erythema	27/49 (55.1%)	18/29 (62.1%)	9/20 (45.0%)	
Based on extent of skin with raised temps	19/49 (38.8%)	14/29 (48.3%)	5/20 (25.0%)	
Based on amount and type of wound exudate	21/49 (42.9%)	14/29 (48.3%)	7/20 (35.0%)	
Based on extent of swelling	20/49 (40.8%)	15/29 (51.7%)	5/20 (25.0%)	
Based on degree of tissue necrosis	30/49 (61.2%)	19/29 (65.5%)	11/20 (55.0%)	
Others	18/49 (36.7%)	6/29 (20.7%)	12/20 (60.0%)	
**Wound sampling prior to surgical treatment**				
Tissue or bone biopsy	17/49 (34.7%)	4/29 (13.8%)	13/20 (65.0%)	*p* < 0.001 ^3^
Wound swab	27/49 (55.1%)	22/29 (75.9%)	5/20 (25.0%)	
Others	5/49 (10.2%)	3/29 (10.3%)	2/20 (10.0%)	
**Guideline usage**				
Yes	14/49 (28.6%)	10/29 (34.5%)	4/20 (7.4%)	*P* = 0.435 ^4^
No	35/49 (71.4%)	19/29 (65.5%)	16/20 (29.6%)	
**Antibiotic choice**				
Piperacillin/Tazobactam	21/41 (51.2%)	13/22 (59.1%)	8/19 (42.1%)	*p* = 0.871 ^3^
Amoxicillin/Clavulanic acid	8/41 (19.5%)	4/22 (18.2%)	4/19 (21.1%)	
Cefazolin	5/41 (12.2%)	2/22 (9.1%)	3/19 (15.8%)	
Defer to guidelines or infectious diseases	5/41 (12.2%)	2/22 (9.1%)	3/19 (15.8%)	
Other antibiotics	2/41 (4.9%)	1/22 (4.5%)	1/19 (5.3%)	
**Antibiotic route**				
IV	31/40 (77.5%)	18/21 (85.7%)	13/19 (68.4%)	*p* = 0.518 ^3^
IV + Oral	2/40 (5.0%)	1/21 (4.8%)	1/19 (5.3%)	
Defer to guidelines or infectious diseases physicians	5/40 (12.5%)	2/21 (9.5%)	3/19 (15.8%)	
Others	2/40 (5.0%)	0/21 (0.0%)	2/19 (10.5%)	
**Wound dressing selection**^**1**^				
Iodine-based dressings	26/42 (61.9%)	14/22 (63.6%)	12/20 (60.0%)	^2^
Betadine paint	10/42 (23.8%)	9/22 (40.9%)	1/20 (5.0%)	
Saline soaked packing	19/42 (45.2%)	12/22 (54.5%)	7/20 (35.0%)	
Betadine soaked packing	13/42 (31.0%)	10/22 (45.5%)	3/20 (15.0%)	
Chlorohexidine-based dressings	1/42 (2.4%)	0/22 (0.0%)	1/20 (5.0%)	
Silver-based dressings	23/42 (54.8%)	11/22 (50.0%)	12/20 (60.0%)	
Honey-based dressings	1/42 (2.4%)	0/22 (0.0%)	1/20 (5.0%)	
Negative pressure therapy	38/42 (90.5%)	19/22 (86.4%)	19/20 (95.0%)	
No dressing	2/42 (4.8%)	2/22 (9.1%)	0/20 (0.0%)	
Others	9/42 (21.4%)	8/22 (36.4%)	1/20 (5.0%)	
**Wound closure after debridement**^**1**^				
Healing by primary closure	19/42 (45.2%)	10/22 (45.5%)	9/20 (45.0%)	^2^
Healing by delayed primary closure	27/42 (64.3%)	13/22 (59.1%)	14/20 (70.0%)	
Superficial skin graft	18/42 (42.9%)	13/22 (59.1%)	5/20 (25.0%)	
Healing by secondary intention	39/42 (92.9%)	20/22 (90.9%)	19/20 (95.0%)	

^1^% do not add up to 100% as participants could select multiple responses, ^2^ As responders could indicate a positive response to more than one option statistical testing was not possible due to the dependence of responses, ^3^ Fisher-Freeman-Halton test, ^4^ Yates continuity correction

Respondents who used guidelines were also asked to detail what they thought was lacking in the current guidelines. Three main themes were identified from their responses: (i) Treatment-based decisions, (ii) Holistic management and (iii) Local resource considerations. Table 3 reports this in greater detail.

**Table 3:** Additional areas to be covered in guidelines

Additional areas to be covered in guidelines	n
Treatment-based decisions • *Indications for conservative vs. surgical management* • *Optimal offloading and biomechanical considerations* • *Vascular intervention* • *Imaging criteria* • *Charcot vs. infection considerations*	9
Holistic management • *Co-morbidities* • *Indicators of function* • *Patient education* • *Dietary management of diabetes*	7
Local resource consideration • *Distance to nearest hospital* • *Availability of podiatry services*	2

Sample quotes are given below to illustrate these themes:

(i) Treatment-based decisions.

*“The timing of debridement is not clear” (Orthopaedic surgeon 11)*.

*“The role of total contact casting. Determining infection vs Charcot arthropathy.” (Orthopaedic surgeon 7)*.

(ii) Holistic management.

*“Adding patients’ baseline levels (ADSLs)… life expectancy and other co-morbidities into equation” (Vascular surgeon 21)*.

*“Long-term indicators of function (i.e. cognitive assessment; health literacy; social networking)” (Vascular surgeon 15)*.

(iii) Local resource considerations.

*“Consideration of local resources available like podiatry services.” (Orthopaedic surgeon 17)*.

Half (21/41; 51.2%) of the respondents indicated that piperacillin combined with tazobactam was their preferred choice of antibiotic for the empirical management of severe DFI. Most (31/40; 77.5%) respondents preferred an intravenous route of antibiotic administration. Few respondents indicated that their choice (5/41; 12.2%) and route (5/40; 12.5%) of antibiotic was based on guidelines or on advice from infectious diseases physicians. There was no statistical difference in the choice and route of delivery for antibiotics between orthopaedic and vascular surgery specialties (Fisher-Freeman-Halton = 1.799, *p* = 0.871; Fisher-Freeman-Halton = 2.832, *p* = 0.518). Negative pressure dressings were the most common method used to manage open wounds (38/42; 90.5%) with most wounds left to heal by secondary intention (39/42; 92.9%).

### Opinions regarding clinical management and perceptions of further research

Table 4 summarises respondents’ opinions about the usefulness of diagnostic modalities for osteomyelitis, variation in clinical practice, confidence in managing key aspects of DFI and perceptions on the need for further clinical trials. Results were reported as median (IQR). The probe-to-bone test (4/5 [3–5]) and magnetic resonance imaging (4/5 [3–5]) were seen as the most useful ways to diagnose osteomyelitis and this was not significantly different between the vascular and orthopaedic surgeons (*p* = 0.082, *p* = 0.922). Respondents were confident about making management decisions with a median confidence score of 4 out of 5 in all aspects covered. Respondents indicated greatest confidence in the indications for surgical debridement (5/5 [4, 5]). Respondents felt that there was moderate variation (i.e. median variation score of 3) between specialists in most management decisions. The choice of wound dressing was felt to be particularly variable (4/5 [3–5]). Respondents perceived moderate need (minimum median score of 3) for further randomised controlled trials exploring key aspects of DFI management. There was no statistically significant difference between the response of vascular and orthopaedic surgeons.

**Table 4:** Opinions of managing diabetes-related foot infection and osteomyelitis

	Total	Vascular surgeons	Orthopaedic surgeons	*p*-value ^2^
**Usefulness for diagnosing diabetic foot osteomyelitis**^**1**^				
Probe-to-bone test	35/49 4 (3–5)	19/29 4 (3–5)	16/20 3 (2–4)	*p *= 0.082
Bone biopsy	35/49 3 (2–5)	18/29 3 (2–4)	17/20 4 (2–5)	*p *= 0.303
Plain x-ray	40/49 3 (2–4)	22/29 3 (2–4)	18/20 3.5 (2–4)	*p* = 0.757
Magnetic resonance imaging	39/49 4 (3–5)	21/29 4 (3.5–5)	18/20 4 (3–5)	*p* = 0.922
Bone scan	24/49 3 (2–4)	12/29 3 (2–4)	12/20 3 (2–3.75)	*p* = 0.478
PET-CT scan	19/49 3 (3–4)	10/29 3 (2.75–4)	9/20 4 (3–4)	*p* = 0.720
**Confidence in**: ^**1**^				
Wound dressing choice	40/49 4 (3.25–5)	21/29 5 (4–5)	19/20 4 (3–4)	*P* = 0.065
Antibiotic choice	39/49 4 (4–5)	21/29 4 (4–5)	18/20 4 (3–4)	*P* = 0.053
Antibiotic duration	40/49 4 (3–4)	22/29 3.5 (3–4)	18/20 4 (3–4)	*P* = 0.638
Indications for removal of infected bone	41/49 4 (4–5)	22/29 4.5 (4–5)	19/20 4 (4–5)	*P* = 0.954
Indications for surgical debridement	42/49 5 (4–5)	22/29 5 (4–5)	20/20 4 (4–5)	*P* = 0.083
Extent of surgical debridement	42/49 4 (4–5)	22/29 5 (4–5)	20/20 4 (4–5)	*P* = 0.190
**Variation in**: ^**1**^				
Wound dressing choice	41/49 4 (3–5)	21/29 4 (3.5–5)	20/20 4 (3–4)	*p* = 0.122
Antibiotic choice	40/49 3 (2–4)	21/29 3 (2–3.5)	19/20 3 (2–4)	*p* = 0.830
Antibiotic duration	40/49 3 (2–3.75)	21/29 3 (3–4)	19/20 3 (2–4)	*P* = 0.320
Indications for removal of infected bone	38/49 3 (3–4)	19/29 3 (2–3)	19/20 3 (2–4)	*P* = 0.525
Indications for surgical debridement	38/49 3 (2–4)	20/29 3 (2–4)	18/20 3 (2–4)	*P* = 0.806
Extent of surgical debridement	41/49 3 (2–4)	21/29 3 (2–4)	20/20 3 (2–4)	*P* = 0.764
**Need for further randomised clinical trials exploring**: ^**1**^				
Wound dressing choice	31/49 4 (3–4)	16/29 4 (3–5)	15/20 3 (3–4)	*p* = 0.216
Antibiotic choice	33/49 3 (2–4)	18/29 3 (2–4)	15/20 3 (2–4)	*p* = 0.789
Antibiotic duration	35/49 4 (3–4)	18/29 4 (3–4)	17/20 4 (2.5–4)	*p* = 0.732
Indications for removal of infected bone	34/49 4 (3–5)	17/29 4 (3–5)	17/20 4 (2.5–5)	*p* = 0.610
Indications for surgical debridement	32/49 4 (2–5)	14/29 4 (2–4.25)	18/20 4 (2.75–5)	*p* = 0.639
Extent of surgical debridement	34/49 4 (2.75–4.25)	16/29 4 (3–4.75)	18/20 4 (2–4.25)	*p* = 0.506

^1^ Reported as median (IQR), ^2^ Mann-Whitney U test

In an optional open-ended question, respondents were asked about their management decision for revascularisation. Content analysis identified two main themes: (i) Revasularisation decision based on assessment of the severity of ischemia and (ii) Referral to vascular surgery for revasularisation decisions. Table 5 reports this in greater detail.

**Table 5:** Revascularisation decisions

Revascularisation decision	Total	Vascular surgery	Orthopaedic surgery
Severity of ischemia determined via: • Toe pressures/ABI ^1^ • Palpation of foot/lower limb pulses • Imaging modalities • Wound healing & appearance	23/33 (69.7%)	18/18 (100.0%)	5/15 (33.3%)
Referral to vascular surgery	10/33 (30.3%)	0/18 (0%)	10/15 (66.7%)

^1^ ABI = Ankle brachial pressure index

Sample quotes are given below:

(i) Severity of ischemia.

This theme formed the largest section of comments (23/33; 69.7%). All vascular surgeons who commented (18/18; 100.0%) on this question mentioned that they would make revascularisation decisions based on the severity of ischemia. These were determined using a combination of imaging modalities, ankle and toe Doppler pressures, palpation of pulses and/or based on the healing and appearance of the wound itself.

*“Clinical assessment along with aid of toe pressures, duplex ultrasound and addition of MRA or angiography.” (Vascular surgeon 29)*.

*“Degree of ischemia of tissues clinically, absent pulses and toe pressures.” (Vascular surgeon 22)*.

(ii) Referral to vascular surgery.

In contrast, a majority of orthopaedic surgeons who commented on this question mentioned that they would refer to vascular surgery to make revascularisation decisions (10/15; 66.7%).

*“Vascular consults, guided by their (vascular surgery) valued opinion – if they believe there is a benefit we run with it.” (Orthopaedic surgeon 15)*.

### Private vs. public setting differences

The responses of surgeons that mainly worked in public hospitals were not significantly different to those mainly working in the private sector, with one exception (Additional File 2). Surgeons working privately were more likely to see value (4/5 on a 5-point Likert rating scale) in RCTs testing different wound dressings compared to those working in the public setting (3/5) (*p* = 0.040).

## Discussion

This study is the first to report the opinions of surgeons about management of severe DFI. The main finding was that relatively few vascular and orthopaedic surgeons felt guidelines were valuable in guiding decisions on DFI management. Vascular and orthopedic surgeons did not defer significantly in their responses on most management aspects such as the type of empirical antibiotic, method to diagnose DFO and dressing selection. There was a notable difference in the wound sampling method preferred to guide choice of antibiotic. Vascular surgeons were more inclined to use wound swabs compared to orthopaedic surgeons who preferred tissue or bone biopsies. The reason for this variation is not clear and might warrant further investigation.

Currently, best-practice guidelines recommend a tissue or bone biopsy as gold standard in determining the causative pathogen for osteomyelitis [3]. Bone biopsy is invasive and it is likely vascular surgeons may have felt it inappropriate to perform a bone biopsy unless an amputation was being performed as part of clinical care. Less than one-third of respondents indicated they used clinical guidelines to guide their management of DFI. The survey did not gather information on the reasons for this, though one possible explanation is the lack of focused guidelines on the surgical management of DFI. For example, the current IWGDF guidelines include only a handful of recommendations on the surgical management of DFI [3]. A number of respondents indicated in their free text responses that guidelines could be improved by covering specific treatment decisions and providing a more holistic management approach. Past research suggest that barriers to use of best-practice guidelines for wound care include the heterogeneous presentation of DFI, complexity of best-practice recommendations and lack of financial incentives to following best practice [12, 13]. Warriner and Carter in their review of wound care guidelines suggested that these could be advanced by greater patient involvement and more complete consideration of the effect of varying resources on implementation, such as the impact of a tertiary hospital versus a regional setting [13]. Currently, the International Working Group on the Diabetic Foot (IWGDF) guidelines are recommended for clinical management of DFI in Australia [11]. It is likely that the IWGDF guideline, although evidence-based, lacks contextualisation to the local situation and is therefore not widely adopted, as evidenced in our survey. Work is currently being undertaken to develop up-to-date Australian-specific guidelines for diabetes-related foot management using the IWGDF guidelines as a reference [14]. The development of the Australian-specific guidelines has undergone rigorous initial public consultations and review by local experts, with an up-to-date Australian guideline expected to be released later this year [14]. It is also important to note the lack of randomised controlled trials testing different surgical approaches for DFI means that current guidelines lack high-quality evidence to inform recommendations. The lack of large randomised controlled trials to inform best management of diabetes-related foot disease makes it challenging to provide  recommendations.

Survey respondents agreed on the usefulness of the probe-to-bone test and magnetic resonance imaging (MRI) in diagnosing osteomyelitis. This is in line with past research and current guidelines [15–17]. Although survey respondents were confident in managing DFI, they indicated there was a moderate variation in management, particularly in relation to choice of wound dressing. Given the myriad of wound dressings available and the varying costs associated with different types of wound dressings this is not surprising. Currently, there is no robust evidence suggesting superiority of one dressing over another and best-practice guidelines suggest the choice of dressings should be based on wound healing principles, dressing costs and patient preferences. For treatment of infected wounds, dressings should contain antimicrobial properties [18, 19]. For management of a post-surgical diabetes-related foot wound, current guidelines suggest considering the use of negative pressure wound therapy [19].

Similar to this study a survey amongst Australian and New Zealand infectious diseases physicians suggested they preferred using MRI to diagnose osteomyelitis and reported limited use of clinical guideline to aid management of DFI [9].The majority of infectious diseases physicians preferred using superficial swabs to guide the use of antibiotics. The heterogeneity of antimicrobial treatments reported in the survey of infectious diseases physicians was less evident in the current survey, with approximately half of respondents indicating piperacillin combined with tazobactam was the preferred antibiotic. A possible explanation for this disparity is that the current survey asked about the initial empirical antibiotic management of DFI and thus it was expected that respondents would list an antibiotic that provides a broad spectrum of antimicrobial cover.

Survey respondents identified a moderate need for further clinical trials testing key aspects of management. This is in keeping with the IWGDF guidelines and a recently published systematic review, which have highlighted uncertainties in many areas of managing DFI due to the lack of high-quality clinical trials [3, 6]. Surgeons that mainly worked in the private sector perceived greater value in randomised clinical trials testing wound dressings than those working at public hospitals. The reasons for this difference are not clear but could relate to greater availability of different types of dressings within the private than public setting due to disparate economic situations.

The strengths of this study include the inclusion of open-ended survey questions, which have provided greater insight into DFI management in vascular and orthopaedic surgeon respondents. This study also had several limitations which should be considered when interpreting the results. Firstly, as the surveys were disseminated through multiple professional societies, it was not possible to determine the response rate. The findings are also subject to participant bias, with respondents expected to be more likely than non-responders to have an interest or expertise in managing DFI. In addition, it was not possible to examine variation in DFI practice, particularly between surgeons working in metropolitan and regional areas as postcodes of respondents were not collected. Also, the survey tool was piloted only amongst vascular and not orthopaedic surgeons, meaning the tool may have not been optimised for orthopaedic surgeons and may have missed answering key questions of this speciality. Over half of the participants were from Queensland, and while there were no differences identified between participants according to state of practice, the results may be less representative of other regions of Australia. Lastly, as the study was conducted in Australia and New Zealand, the findings may not be relevant to other countries.

## Conclusions

In conclusion, this survey suggests that Australian and New Zealand vascular and orthopaedic surgeons have relatively similar management approaches for DFI. A statistically significant difference in the preferred wound sampling method was noted. Few of the responding surgeons used best-practice guidelines to guide management of DFI. There was a perceived moderate variation in clinical management and moderate need for clinical trials investigating key aspects of DFI management. Our findings highlights that the available evidence supporting different treatments and the related guidelines for the surgical managing of DFI need to be advanced.

## Supplementary information

Additional file 1. Containing the survey questions.

Additional file 2. Containing tables comparing private and public setting differences are provided.

## Data Availability

Survey data are available from the corresponding author on reasonable request.

## Abbreviations

DFI: Diabetes-related foot infection
DFO: Diabetes-related foot osteomyelitis
MRI: Magnetic resonance imaging
IWGDF: International Working Group on the Diabetic Foot
RCT: Randomised clinical trials
