# Supplementary material for: Opinions about the most appropriate surgical management of diabetes-related foot infection: a cross-sectional survey
Source: J Foot Ankle Res. 2022 Mar 2;15:18. doi: 10.1186/s13047-022-00523-w (PMC8889647; doi:10.1186/s13047-022-00523-w)
Supplement: Supplementary file 1 — Containing the survey questions. [file 13047_2022_523_MOESM1_ESM.docx]

**Additional File 1:** Survey questions

# Section 1: Background information

1. Please indicate your surgical specialty:
   1. Vascular Surgery
   2. Orthopaedic Surgery
2. Please indicate your role/designation:
   1. RACS accredited Consultant
   2. SET trainee (training scheme)
   3. Unaccredited registrar/ PHO/ junior surgeon
   4. Senior registrar
   5. Registrar
   6. Intern / Resident
   7. Other (please specify): _____________
3. Year of medical qualification:
4. Where is your main practice located?
   1. Queensland
   2. New South Wales
   3. Victoria
   4. South Australia
   5. Tasmania
   6. Northern Territory
   7. Australian Capital Territory
   8. Western Australia
   9. New Zealand; North Island
   10. New Zealand; South Island
   11. Other (please specify): _____________
5. Where is your primary place of work?
   1. Private practice
   2. Public hospital
   3. Non-clinical role (e.g. academic)
   4. Other (please specify): _____________

# Section 2: Diabetes-related foot infection

1. How do you determine the extent of infection within the foot prior to surgical treatment? (**select all that apply**)
   1. Based on an international classification system (please specify which one):
   2. Based on the extent of erythema (i.e. degree of cellulitis)
   3. Based on the extent of skin with raised temperature
   4. Based on the amount and type of exudate from a wound
   5. Based on the extent of swelling
   6. Based on the degree of tissue necrosis
   7. Other (please specify):
2. In patients with diabetic foot infection requiring in-patient treatment **where surgical debridement has not yet been performed**, what method of wound sampling do you most commonly use to guide antibiotic choice?
3. Tissue or bone biopsy
4. Wound aspirate
5. Wound swab
6. Other (please specify):
7. Do you use guidelines to base your management of diabetic foot infection requiring in-patient treatment?
   1. Yes, (which guideline:______)
   2. No *(skip question 9 if this option is selected)*
8. How useful is the guideline you have specified in question 8 for assisting in the decision making regarding surgical management of diabetic foot infection? (where **1 is least useful** and **5 is most useful**)

|  | Least useful |  |  |  | Most useful |
| --- | --- | --- | --- | --- | --- |

|  | 1 | 2 | 3 | 4 | 5 |
| --- | --- | --- | --- | --- | --- |

1. What additional areas should be covered in the guidelines for assisting in the decision making regarding surgical management of diabetic foot infection?
2. How do you decide whether revascularisation is needed in a patient with diabetic foot infection requiring debridement?
3. In a patient with an infected diabetic foot ulcer who has undergone extensive debridement for source control, how frequently do you use the following approaches for wound closure? (Must total 100%)
   1. Healing by primary closure (%): _______
   2. Healing by delayed primary closure (%): _______
   3. Superficial skin graft to expedite closure (%): _______
   4. Healing by secondary intention with the assistance of a VAC dressing (%): _______
4. How frequently do you use the following antiseptic dressings for the **in-patient** management of a diabetic foot infection? (Must total 100%)
   1. Iodine-based dressings (i.e iodosorb, betadine) (%): _______
   2. Betadine paint and left to air dry (%): _______
   3. Saline soaked packing (%): _______
   4. Betadine soaked packing (%): _______
   5. Chlorohexidine-based dressings, if so which one? (%): _______
   6. Silver-based dressings, if so which one? (%): _______
   7. Honey-based dressings, if so which one? (%): _______
   8. VAC dressings (%): _______
   9. No dressing (%): _______
   10. Other (please specify) (%): _______
5. For **initial** **empirical** management of diabetic foot infection requiring in-patient treatment, which antibiotic regimen and route do you use?
   Antibiotic:

Dose:
Route:

1. Within your practice, of the patients admitted to hospital for the management of a diabetic foot infection, what percentage do not require surgical debridement (in %)?

# Section 3: Diabetes-related foot osteomyelitis

1. In a patient who you suspect has diabetic foot osteomyelitis, how useful do you find these tests in reaching a diagnosis? (Where **1 = least useful** and **5 = most useful**)

|  | \|  \| Least useful \|  \|  \|  \| Most useful \| \| --- \| --- \| --- \| --- \| --- \| --- \| | | | | |
| --- | --- | --- | --- | --- | --- | --- | --- | --- | --- | --- | --- |
| Probing to bone | 1 | 2 | 3 | 4 | 5 |
| Bone biopsy | 1 | 2 | 3 | 4 | 5 |
| Plain x-ray | 1 | 2 | 3 | 4 | 5 |
| MRI | 1 | 2 | 3 | 4 | 5 |
| Bone scan | 1 | 2 | 3 | 4 | 5 |
| PET-CT scan | 1 | 2 | 3 | 4 | 5 |
| Other (please specify) | 1 | 2 | 3 | 4 | 5 |

1. In patients with diabetic foot osteomyelitis that you treat, how frequently do you successfully avoid surgical removal of the infected bone (i.e. treat with antibiotics alone)? (where **1 = least frequent** and **5 = most frequent**)

| Least frequent |  | Most frequent |
| --- | --- | --- |

| 1 | 2 | 3 | 4 | 5 |
| --- | --- | --- | --- | --- |

# Section 4: Clinical consensus and further research needed

1. How confident do you feel about making the following management decisions in all the patients you treat with diabetic foot infection (where **1 = not very confident** and **5 = very confident**):

|  | \|  \| Not very confident \|  \|  \|  \| Very confident \| \| --- \| --- \| --- \| --- \| --- \| --- \| | | | | |
| --- | --- | --- | --- | --- | --- | --- | --- | --- | --- | --- | --- |
| Choice of wound dressing | 1 | 2 | 3 | 4 | 5 |
| Choice of antibiotics | 1 | 2 | 3 | 4 | 5 |
| Duration of antibiotics | 1 | 2 | 3 | 4 | 5 |
| Indications for removal of infected bone | 1 | 2 | 3 | 4 | 5 |
| When surgical debridement is required | 1 | 2 | 3 | 4 | 5 |
| Extent of surgical debridement required | 1 | 2 | 3 | 4 | 5 |

1. How much variation in practice between surgeons do you believe the following management decisions about patients with diabetic foot infection are? (where **1 = no variation** **between surgeons** and **5 = enormous variation between surgeons**)

|  | \|  \| No variation between surgeons \|  \|  \|  \| Enormous variation between surgeons \| \| --- \| --- \| --- \| --- \| --- \| --- \| | | | | |
| --- | --- | --- | --- | --- | --- | --- | --- | --- | --- | --- | --- |
| Choice of wound dressing | 1 | 2 | 3 | 4 | 5 |
| Choice of antibiotics | 1 | 2 | 3 | 4 | 5 |
| Duration of antibiotics | 1 | 2 | 3 | 4 | 5 |
| Indications for removal of infected bone | 1 | 2 | 3 | 4 | 5 |
| When surgical debridement is required | 1 | 2 | 3 | 4 | 5 |
| Extent of surgical debridement required | 1 | 2 | 3 | 4 | 5 |

1. In your opinion, which management aspects should be addressed in a randomised controlled trial for diabetic foot infection and/or diabetic foot osteomyelitis? (where **1 = no value to investigate in an RCT** and **5 = very valuable to investigate in an RCT**)

|  | \|  \| No value to investigate in RCT \|  \|  \|  \| Very valuable to investigate in RCT \| \| --- \| --- \| --- \| --- \| --- \| --- \| | | | | |
| --- | --- | --- | --- | --- | --- | --- | --- | --- | --- | --- | --- |
| Choice of wound dressing | 1 | 2 | 3 | 4 | 5 |
| Choice of antibiotics | 1 | 2 | 3 | 4 | 5 |
| Duration of antibiotics | 1 | 2 | 3 | 4 | 5 |
| Indications for removal of infected bone | 1 | 2 | 3 | 4 | 5 |
| When surgical debridement is required | 1 | 2 | 3 | 4 | 5 |
| Extent of surgical debridement required | 1 | 2 | 3 | 4 | 5 |

1. Would you be interested in taking part in a randomised controlled trial of management for diabetic foot infection and/or diabetic foot osteomyelitis?
   1. No
   2. Yes (please provide your email address and other relevant contact details below)
