# Supplementary material for: Opinions about the most appropriate surgical management of diabetes-related foot infection: a cross-sectional survey
Source: J Foot Ankle Res. 2022 Mar 2;15:18. doi: 10.1186/s13047-022-00523-w (PMC8889647; doi:10.1186/s13047-022-00523-w)
Supplement: Supplementary file 2 — Containing tables comparing private and public setting differences are provided. [file 13047_2022_523_MOESM2_ESM.docx]

**Additional File 2:** Comparing Private vs Public setting differences

**Supplementary Table 1:** Current management practice (Private vs Public setting)

|  | **Total** | **Private** | **Public** | **p-value** |
| --- | --- | --- | --- | --- |
| **Determining extent of infection prior to surgical treatment ^1^** | | | | |
| Based on international classification system | 13/49 (26.5%) | 5/18 (27.8%) | 8/31 (25.8%) | ^2^ |
| Based on extent of erythema | 27/49 (55.1%) | 9/18 (50.0%) | 18/31 (58.1%) |  |
| Based on extent of skin with raised temps | 19/49 (38.8%) | 6/18 (33.3%) | 13/31 (41.9%) |  |
| Based on amount and type of wound exudate | 21/49 (42.9%) | 7/18 (38.9%) | 14/31 (45.2%) |  |
| Based on extent of swelling | 20/49 (40.8%) | 6/18 (33.3%) | 14/31 (45.2%) |  |
| Based on degree of tissue necrosis | 30/49 (61.2%) | 10/18 (55.6%) | 20/31 (64.5%) |  |
| Others | 18/49 (36.7%) | 6/18 (33.3%) | 12/31 (38.7%) |  |
| **Wound sampling prior to surgical treatment** | | | | |
| Tissue or bone biopsy | 17/49 (34.7%) | 5/18 (27.8%) | 12/31 (38.7%) | 0.760 ^3^ |
| Wound swab | 27/49 (55.1%) | 11/18 (61.1%) | 16/31 (51.6%) |  |
| Others | 5/49 (10.2%) | 2/18 (11.1%) | 3/31 (9.7%) |  |
| **Guideline usage** | | | | |
| Yes | 14/49 (28.6%) | 4/18 (22.2%) | 10/31 (32.3%) | 0.673 ^4^ |
| No | 35/49 (71.4%) | 14/18 (77.8%) | 21/31 (67.7%) |  |
| **Antibiotic choice** | | | | |
| Piperacillin/Tazobactam | 21/41 (51.2%) | 11/15 (73.3%) | 10/26 (38.5%) | 0.202 ^3^ |
| Amoxicillin/Clavulanic acid | 8/41 (19.5%) | 1/15 (6.7%) | 7/26 (26.9%) |  |
| Cefazolin | 5/41 (12.2%) | 1/15 (6.7%) | 5/26 (19.2%) |  |
| Defer to guidelines or infectious diseases | 5/41 (12.2%) | 2/15 (13.3%) | 3/26 (11.5%) |  |
| Other antibiotics | 2/41 (4.9%) | 0/15 (0.0%) | 1/26 (3.8%) |  |
| **Antibiotic route** | | | | |
| IV | 31/40 (77.5%) | 13/16 (81.3%) | 18/24 (75.0%) | 0.910 ^3^ |
| IV + Oral | 2/40 (5.0%) | 1/16 (6.3%) | 1/24 (4.2%) |  |
| Defer to guidelines or infectious diseases physicians | 5/40 (12.5%) | 2/16 (12.5%) | 3/24 (12.5%) |  |
| Others | 2/40 (5.0%) | 0/16 (0.0%) | 2/24 (8.3%) |  |
| **Wound dressing selection ^1^** | | | | |
| Iodine-based dressings | 26/42 (61.9%) | 9/16 (56.3%) | 17/26 (65.4%) | ^2^ |
| Betadine paint | 10/42 (23.8%) | 3/16 (18.8%) | 7/26 (26.9%) |  |
| Saline soaked packing | 19/42 (45.2%) | 9/16 (56.3%) | 10/26 (38.5%) |  |
| Betadine soaked packing | 13/42 (31.0%) | 3/16 (18.8%) | 10/26 (38.5%) |  |
| Chlorohexidine-based dressings | 1/42 (2.4%) | 1/16 (6.3%) | 0/26 (0.0%) |  |
| Silver-based dressings | 23/42 (54.8%) | 10/16 (62.5%) | 13/26 (50.0%) |  |
| Honey-based dressings | 1/42 (2.4%) | 0/16 (0.0%) | 1/26 (3.8%) |  |
| Negative pressure therapy | 38/42 (90.5%) | 14/16 (87.5%) | 24/26 (92.3%) |  |
| No dressing | 2/42 (4.8%) | 0/16 (0.0%) | 2/26 (7.7%) |  |
| Others | 9/42 (21.4%) | 4/16 (25.0%) | 5/26 (19.2%) |  |
| **Wound closure after debridement ^1^** | | | | |
| Healing by primary closure | 19/42 (45.2%) | 5/16 (31.3%) | 14/26 (53.8%) | ^2^ |
| Healing by delayed primary closure | 27/42 (64.3%) | 11/16 (68.8%) | 16/26 (61.5%) |  |
| Superficial skin graft | 18/42 (42.9%) | 7/16 (43.8%) | 11/26 (42.3%) |  |
| Healing by secondary intention | 39/42 (92.9%) | 14/16 (87.5%) | 25/26 (96.2%) |  |

^1^ % do not add up to 100% as participants could select multiple responses, ^2^ As responders could indicate a positive response to more than one option statistical testing was not possible due to the dependence of responses, ^3^ Halton-Freeman extension of Fisher’s exact test, ^4^ Yates continuity correction

**Supplementary Table 2:** Perceptions of managing diabetes-related foot infection and osteomyelitis (Private vs Public setting)

|  | **Total** | **Private** | **Public** | **p-value** ^2^ |
| --- | --- | --- | --- | --- |
| **Usefulness for diagnosing diabetic foot osteomyelitis ^1^** | | | | |
| Probe-to-bone test | 35/49 4 (3 – 5) | 13/18 4 (2.5 – 4) | 22/31 4 (2 – 5) | 0.578 |
| Bone biopsy | 35/49 3 (2 – 5) | 12/18 4 (3 – 5) | 23/31 3 (2 – 4) | 0.057 |
| Plain x-ray | 40/49 3 (2 – 4) | 14/18 3 (2 – 4) | 26/31 3.5 (2 – 4) | 0.856 |
| Magnetic resonance imaging | 39/49 4 (3 – 5) | 16/18 4 (2.25 – 4.75) | 23/31 4 (4 – 5) | 0.187 |
| Bone scan | 24/49 3 (2 – 4) | 9/18 3 (3 – 3.5) | 15/31 2 (2 – 4) | 0.519 |
| PET-CT scan | 19/49 3 (3 – 4) | 7/18 4 (3 – 4) | 12/31 3 (2.25 – 4) | 0.482 |
| **Confidence in: ^1^** | | | | |
| Wound dressing choice | 40/49 4 (3.25 – 5) | 16/18 4 (4 – 5) | 24/31 4 (3 – 5) | 0.374 |
| Antibiotic choice | 39/49 4 (4 – 5) | 15/18 4 (4 – 5) | 24/31 4 (3.25 – 4.75) | 0.809 |
| Antibiotic duration | 40/49 4 (3 – 4) | 14/18 3.5 (3 – 4) | 26/31 4 (3 – 5) | 0.305 |
| Indications for removal of infected bone | 41/49 4 (4 – 5) | 16/18 4 (4 – 5) | 25/31 5 (4 – 5) | 0.179 |
| Indications for surgical debridement | 42/49 5 (4 – 5) | 16/18 4.5 (4 – 5) | 26/31 5 (4 – 5) | 0.505 |
| Extent of surgical debridement | 42/49 4 (4 – 5) | 16/18 4 (4 – 5) | 26/31 5 (4 – 5) | 0.224 |
| **Variation in: ^1^** | | | | |
| Wound dressing choice | 41/49 4 (3 – 5) | 15/18 4 (4 – 5) | 26/31 4 (3 – 5) | 0.738 |
| Antibiotic choice | 40/49 3 (2 – 4) | 14/18 3 (2 – 3.25) | 26/31 3 (2 – 4) | 0.644 |
| Antibiotic duration | 40/49 3 (2 – 3.75) | 14/18 3 (2 – 4) | 26/31 3 (3 – 4) | 0.424 |
| Indications for removal of infected bone | 38/49 3 (3 – 4) | 13/18 (2 – 3.5) | 25/31 3 (2 – 4) | 0.671 |
| Indications for surgical debridement | 38/49 3 (2 – 4) | 13/18 2 (2 – 3) | 25/31 3 (2 – 4) | 0.234 |
| Extent of surgical debridement | 41/49 3 (2 – 4) | 15/18 3 (2 – 4) | 26/31 3 (2 – 4) | 0.925 |
| **Need for further randomised clinical trials exploring: ^1^** | | | | |
| Wound dressing choice | 31/49 4 (3 – 4) | 11/18 4 (4 – 5) | 20/31 3 (2.25 – 4) | 0.040 |
| Antibiotic choice | 33/49 3 (2 – 4) | 11/18 4 (3 – 4) | 22/31 3 (2 – 4) | 0.143 |
| Antibiotic duration | 35/49 4 (3 – 4) | 13/18 4 (3 – 4) | 22/31 4 (2 – 4) | 0.428 |
| Indications for removal of infected bone | 34/49 4 (3 – 5) | 13/18 4 (3 – 5) | 21/31 4 (2 – 4.5) | 0.205 |
| Indications for surgical debridement | 32/49 4 (2 – 5) | 13/18 4 (3.5 – 4.5) | 19/31 4 (2 – 5) | 0.677 |
| Extent of surgical debridement | 34/49 4 (2.75 – 4.25) | 13/18 4 (3 – 5) | 21/31 4 (2 – 4) | 0.292 |

^1^ Reported as median (IQR), ^2^ Mann-Whitney U test
